# A Dengue Virus Type 4 Model of Disseminated Lethal Infection in AG129 Mice

**DOI:** 10.1371/journal.pone.0125476

**Published:** 2015-05-04

**Authors:** Gregg N. Milligan, Vanessa V. Sarathy, Ernesto Infante, Li Li, Gerald A. Campbell, P. Robert Beatty, Eva Harris, Alan D. T. Barrett, Nigel Bourne

**Affiliations:** 1 Department of Pediatrics, University of Texas Medical Branch, Galveston, Texas, United States of America; 2 Sealy Center for Vaccine Development, University of Texas Medical Branch, Galveston, Texas, United States of America; 3 Department of Microbiology and Immunology, University of Texas Medical Branch, Galveston, Texas, United States of America; 4 Department of Pathology, University of Texas Medical Branch, Galveston, Texas, United States of America; 5 Division of Infectious Diseases and Vaccinology, School of Public Health, University of California, Berkeley, California, United States of America; 6 Department of Molecular and Cell Biology, University of California, Berkeley, California, United States of America; University of Rochester, UNITED STATES

## Abstract

Dengue is a mosquito-borne disease of global public health significance that is caused by four serologically and genetically related viruses (DENV-1 to DENV-4). Most human DENV infections are asymptomatic, but clinical cases can range in severity from a relatively mild self-limiting illness to a severe life-threatening disease. Infection with one serotype of DENV results in life-long homotypic immunity but only short term heterotypic protection. There are no licensed vaccines or antivirals for dengue due in part to difficulty in developing small animal models that mimic the systemic disease seen in humans. Consequently, an important advance was the description of models of DENV-2 infection in AG129 mice (deficient in interferon alpha/beta and gamma receptor signaling) that resemble human disease. However, the need for well characterized models of disease due to DENV-1, -3, and -4 still remains. Here we describe a new AG129 mouse model utilizing a non-mouse-adapted Thai human DENV-4 strain 703-4. Following intraperitoneal challenge, animals experience a rapidly progressive lethal infection without developing neurologic clinical signs of disease. High virus titers are seen in multiple visceral tissues including the liver, spleen and large intestine, and the infected animals develop vascular leakage and thrombocytopenia, hallmarks of human dengue. Taken together, our studies demonstrate that this model is an important addition to the field of dengue research particularly in understanding similarities and differences in the pathologic basis of the disease caused by different DENV serotypes and in determining comparative efficacy of putative vaccines and antivirals.

## Introduction

The genus *Flavivirus* within the family *Flaviviridae* contains viruses that cause a number of important mosquito-borne diseases including yellow fever, Japanese encephalitis, West Nile encephalitis and most importantly dengue, which is caused by four serologically distinct but genetically related viruses (DENV-1 to DENV-4). The World Health Organization (WHO) estimates that approximately 3 billion people currently live in areas where they are at risk of DENV infection and that there are approximately 100 million clinical DENV infections annually worldwide [1, 2]. Recent modelling studies suggest that this may be an underestimate of the current burden of dengue with up to 400 million infections each year [3]. In addition, the disease continues to spread into new geographic areas [4]. Multiple factors are believed to contribute to the increasing public health importance of dengue including expansion of the geographical distribution of two of its principal vectors (*Aedes aegypti* and *Aedes albopictus)*, increases in both urbanization and population size in endemic countries, and growth in international trade and travel.

The majority of human DENV infections are believed to be asymptomatic, but clinical cases range in severity from a relatively mild self-limiting illness to a life-threatening infection. Following the bite of a DENV-infected mosquito, there is typically a 4–10 day incubation period before the onset of clinical symptoms that consist of a flu-like illness, which may be accompanied by a high fever, severe headache, pain in the joints and muscles, nausea, vomiting and rash. The symptoms typically last for 2–7 days before the patient begins to recover. However, in more severe cases the disease progresses with a decrease in temperature accompanied by abdominal pain, persistent vomiting, rapid breathing, plasma leakage, hypovolemic shock, fluid accumulation, respiratory distress, severe bleeding, organ impairment, and occasionally death. These more severe disease manifestations were previously referred to as dengue hemorrhagic fever and dengue shock syndrome, but they have recently been reclassified as severe dengue by the WHO [5]. Recovery from infection with any of the four DENV serotypes confers lifelong homotypic immunity; however, heterotypic protection against the other three serotypes is incomplete and of short duration [6]. Further, evidence suggests that severe dengue can be associated with a second DENV infection due to immune enhancement resulting from either antibody-dependent enhancement (ADE) or cytotoxic cells and/or a cytokine storm [7, 8]. Concerns about the possible role of immune enhancement following heterotypic DENV infection have resulted in the belief that a vaccine against dengue must provide protection against all four DENVs simultaneously.

Despite the clear medical need, there are currently neither licensed vaccines to prevent dengue nor specific therapeutics for treatment. A considerable challenge in this area has been the difficulty of developing animal models that closely recapitulate human disease. Because primates are the only other natural vertebrate hosts of DENVs, non-human primates (NHPs) have been widely studied as models of dengue. Many NHP species have been shown to be susceptible to DENV infection as evidenced by development of viremia with an onset and duration that is similar to that seen in humans [9, 10]. However, in general NHPs develop few signs of disease, which has limited the utility of NHP models [9, 11]. One promising recent exception is that Indian rhesus macaques inoculated intravenously with DENV-2 develop cutaneous hemorrhage [12]. Regardless of the potential utility of this model for coagulopathy, NHPs are not suitable for early phase efficacy testing studies that are more appropriately conducted in small animals. Unfortunately, many small animal species including immunocompetent mice also fail to develop clinical disease following DENV challenge. Thus, a major step forward was the description of a model in AG129 mice (deficient for interferon α/β and γ receptors) in which intraperitoneal (i.p.) inoculation with mouse brain-adapted DENV-2 strain New Guinea C produced a peak viremia on day 3 post-infection (pi), neurological clinical signs on day 7 pi, and death by day 12 pi [13]. However, while humans can experience neurologic involvement during dengue disease, it is not a typical part of the spectrum of clinical presentation of severe dengue. Thus, the impetus to develop a more clinically relevant model remained. Subsequently, a second DENV-2 strain, PL046, that produced lethal neurologic disease in AG129 mice was alternately passaged between mosquito cells and mice to mimic the normal transmission cycle of the virus, and Shresta *et al*. succeeded in generating a new strain, D2S10, that produced a rapidly lethal disseminated disease in AG129 animals without the development of neurologic signs [14]. Studies of D2S10 and the triple-plaque purified derivative strain S221 [15] in AG129 mice have been widely used to explore DEN pathogenesis, understand innate and adaptive immune responses to infection, and to evaluate candidate vaccines and antivirals, e.g. [16–20]. Another recently described lethal AG129 mouse model of disseminated DENV-2 disease utilizes a non-mouse-adapted DENV-2 strain, D2Y98P, derived by passage in mosquito cells from a human Singaporean virus isolate [21, 22].

The fact that AG129 mice are immunodeficient in interferon responses which are important in controlling virus infections imposes limitations on the model. As with all animal models, this means that results must be interpreted carefully in comparing the disease observed in mice to that seen in humans However, in the absence of suitable small animals of dengue in immunocompetent animals the AG129 mouse model has established its value in dengue research and in particular for preclinical evaluation of candidate vaccines and therapeutics.

Until very recently there have been no mouse models for DENV-1, -3 and -4. We have undertaken a series of studies to extend the utility of the AG129 mouse for dengue research and have recently described a DENV-3 model of disseminated disease in AG129 mice [23]. The studies reported here describe our characterization of a model of disseminated lethal DENV-4 disease using a non-mouse-adapted DENV-4 virus.

## Materials and Methods

### Cells and Virus

Monkey kidney Vero cells and mosquito C6/36 cells were obtained from the American Type Culture Collection (ATCC, Manassas, VA). Vero cells were maintained at 37°C in 5% CO_2_ in minimum essential media (MEM) supplemented with 2 mM L-glutamine, 0.1 mM non-essential amino acids, 100 U/ml penicillin—100 μg/ml streptomycin, and 8% bovine growth serum (BGS). C6/36 cells were maintained at 28°C in MEM supplemented with 2 mM L-glutamine, 0.1 mM non-essential amino acids, 100 U/ml penicillin—100 μg/ml streptomycin, 1 mM sodium pyruvate, tryptose phosphate buffer, and 10% fetal bovine serum.

Strain 703–4 is a low passage human isolate collected in Thailand in 1994. Based on Envelope gene sequence it has been classified as a DENV-4 genotype II virus [24]. For these studies, virus stocks were prepared in C6/36 cells, harvested and concentrated using 50K MWCO Amicon filters at 3000 rpm, 4°C, for 20 minutes. Virus stocks were quantified by plaque titration assays in Vero cells. Briefly, Vero cells were infected with 10-fold virus dilutions for 30 minutes before overlay with MEM containing 2% BGS-1% agar and incubated for four days at 37°C. Plaques were counted 2 days post-second overlay with MEM-agar containing 2% neutral red.

### Ethics statement

All animal procedures were reviewed and approved by the Institutional Animal Care and Use Committees of the University of Texas Medical Branch (UTMB) under protocol 03-03-012 and the University of California (UC) Berkeley under protocol R252. The studies were carried out in strict compliance with the recommendations of the Guide for the Care and Use of Laboratory Animals published by the National Research Council.

### Infection of AG129 mice

AG129 (interferon α/β- and γ-receptor-deficient) mice were bred and maintained at animal facilities at UTMB and UC Berkeley. Six-to-eight or 18-week old animals were inoculated by i.p. injection with 7.3 log_10_ pfu DENV-4 703–4 or lower inocula in 0.1 ml volume. Following inoculation, mice were weighed daily and visually monitored and scored for morbidity. Morbidity scoring was based on a 1 to 5 scale: 1-healthy; 2-mild signs of lethargy; 3-lethargy, ruffled fur and hunched posture; 4-lethargy, ruffled fur, hunched posture decreased mobility; 5-moribund. Mice exhibiting signs of severe disease or with weight loss >20% of initial body weight were euthanized. Euthanized animals were counted as being dead on the following day for analysis.

### Generation of mouse anti-DENV sera

Six-to eight week old AG129 mice were infected with 5.0 log_10_ pfu of DENV-2 strain PL046, a dose that causes no clinical signs of disease in the mice but produces a robust antibody response. Six weeks pi mice were euthanized via cardiac puncture and the resultant serum was heat-inactivated and stored frozen (-80^°^C).

### Enhanced DENV infection in AG129 mice

Six-to-eight week old animals were administered either 0.1 ml or 0.2 ml anti-DENV-2 sera or normal mouse serum by i.p. injection. One day later they were infected with a sublethal dose of 6.3 log_10_ pfu DENV-4 703–4 by injection into the tail vein. Following virus infection, animals were monitored for signs of disease as described above.

### Quantitation of virus titers in tissues

On days 1, 2, and 3 pi, AG129 mice were euthanized. Blood was collected by cardiac puncture and serum separated by centrifugation. Samples of liver, spleen, large intestine, and brain tissues were collected into pre-weighed tubes. To determine viral loads, the tissue samples were homogenized using a pestle or a syringe and 16- gauge needle, clarified by centrifugation and then the supernatant titrated in Vero cells by plaque assay as described above.

### Blood chemistry and complete blood count (CBC) determinations

Groups of mice were infected with 7.3 log_10_ pfu DENV-4 703–4 or with medium as uninfected controls. On days 1–3 pi, 3 animals/day/group were sacrificed and blood collected. For blood chemistry determinations, plasma was separated from whole blood by centrifugation and samples were immediately analyzed using a comprehensive diagnostic panel rotor (Vetscan, Abaxis, Union City, CA) according to the manufacturer’s instructions. For CBC determinations, whole blood was treated with EDTA to prevent clotting and samples analyzed immediately using a HEMAVET950TS (Drew Scientific, Dallas TX) according to the manufacturer’s instructions.

### Quantitation of vascular leakage

The method used to measure vascular leakage was based on those described previously [14]. Briefly, groups of mice were infected with 7.3 log_10_ pfu DENV-4 703–4 (N = 6) or served as uninfected controls (N = 7). Two and three days later, 0.2 ml of Evans blue (0.5% solution in PBS) was injected by the intravenous route. After two hours, the mice were euthanized and perfused extensively with PBS. Samples of liver and large intestine were then harvested and placed in pre-weighed tubes containing formamide, incubated for 24 hours, the formamide removed and absorbance at 610 nm measured to determine Evans blue concentration per g tissue weight.

### Histology

Mice were euthanized and liver, spleen, large intestine, and brain samples harvested and immediately fixed in 10% neutral-buffered formalin. The tissues were paraffin embedded, sectioned, and stained with hematoxylin and eosin (H&E) at the UTMB Research Histopathology Core Laboratory. Slides from DENV-4 703-4-infected animals were compared to mock-infected and naive controls to determine changes resulting from virus infection. The liver samples were analyzed for the presence of focal necrosis (FN), nuclear pleomorphism/binucleation of hepatocytes (NP/BN), and estimated glycogen content (G). For hepatocyte glycogen, sections were stained by the periodic acid-Schiff technique.

### Quantification of cytokine and chemokine responses and blood cell counts

Individual serum samples were collected and Bio-Rad Bio-Plex Pro Mouse Cytokine 23-plex used to quantify cytokine levels in 15 ul of serum according to manufacturer’s instructions. Sera collected from mice on days 1–3 pi were analyzed and compared to same-day-matched samples collected from animals inoculated with an equivalent volume of virus-free concentrated tissue culture medium.

### Statistical analyses

Incidence data for infection and disease outcomes were compared by Fisher’s exact test. Student’s t-test or ANOVA were used for comparisons involving group mean values. All p-values presented are two-tailed, with values <0.05 being considered significant. All statistical analysis was performed using GraphPad Prism 5.0 for PC.

## Results

### DENV-4 703–4 produces a lethal infection in AG129 mice

Initial studies evaluated the outcome following i.p. challenge with non-mouse-adapted low passage DENV-4 strain 703–4 in young adult (6-8-week old) AG129 mice. The animals were inoculated with either 7.3 log_10_ pfu (N = 8) or 6.3 log_10_ pfu (N = 8) of the virus and examined daily for morbidity (weight loss and clinical signs) and survival. All of the mice that received the lower virus inoculum survived with no evidence of morbidity, except for a single animal that experienced a transient loss of >10% of its initial body weight.

**Fig 1:**
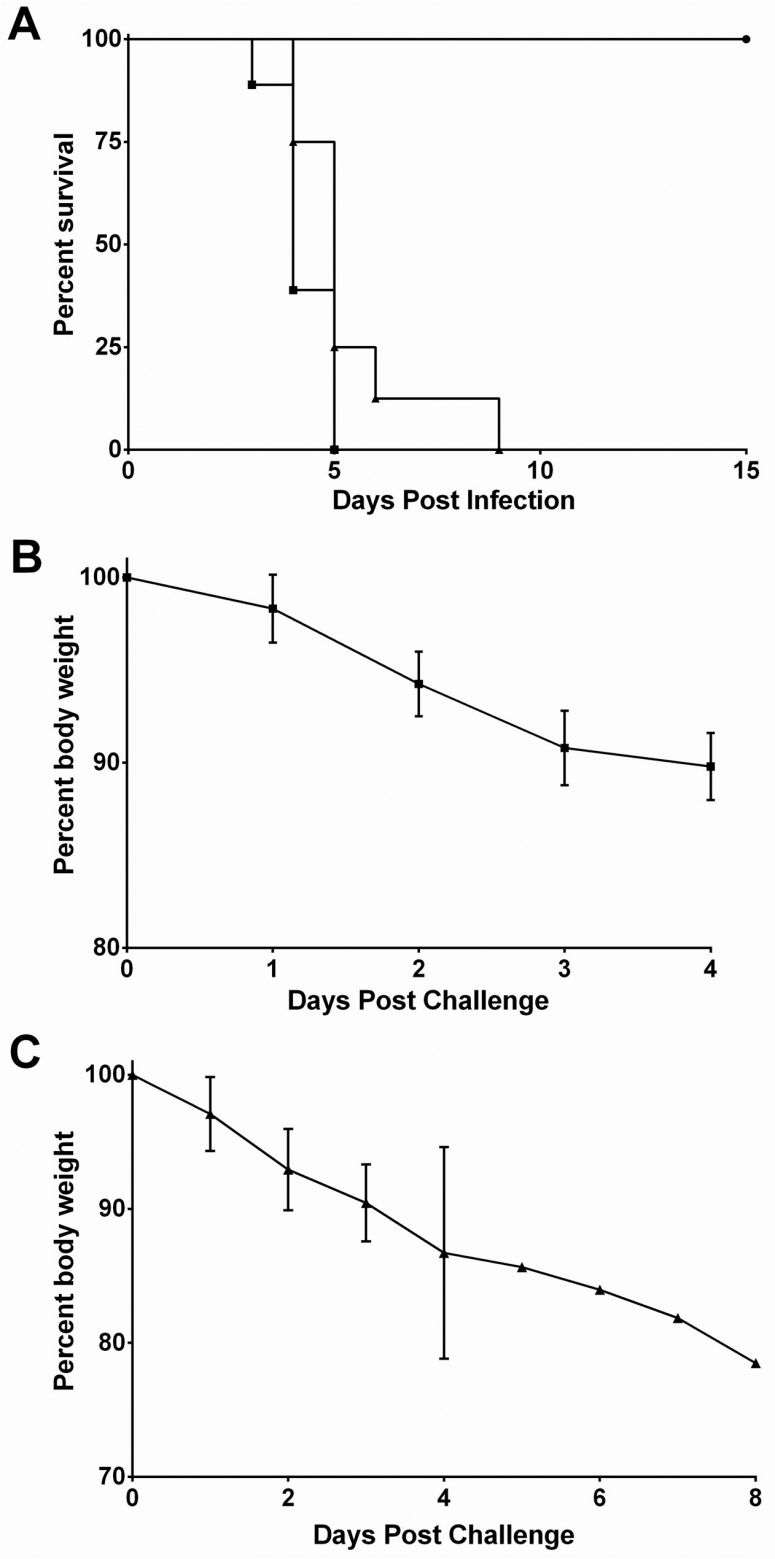
DENV-4 703–4 infection is lethal in AG129 mice. (A) Kaplan-Meier survival curves of 6 week old mice inoculated with 7.3 log_10_ pfu (■; n = 8) or 6.3 log10 pfu (●; n = 8) and 18 week old mice inoculated with 7.3 log_10_ pfu (▲n = 8) DENV-4 703–4 by i.p. injection. Mice were monitored daily for 4 weeks. Morbidity among the 6 week old (B) and 18 week old (C) animals infected with 7.3 log_10_ pfu, as measured by weight loss. Values are mean (±SD) percent weight of initial weight.

In contrast, all of the mice that received the higher dose of virus developed a rapidly progressing disease (Fig 1A). The first sign of this disease was weight loss, which was observed in some animals as soon as day 1pi (Fig 1B). All of the animals had lost weight by day 2 pi (Mean weight 93.5 ± 2.2% initial body weight) and continued weight loss until death. Clinical signs of disease were observed on day 3 pi when mice exhibited ruffled coat, hunched posture, reduced activity, and in some cases, death. There was rapid disease progression with uniform lethality and a mean day of death (MDD) of 4.3 ± 0.7. Interestingly, none of the animals showed any clinical signs of neurologic disease.

To confirm the results with DENV-4 strain 703–4, multiple preparations were generated independently and all confirmed the result above that an inoculum of >7.0 log_10_ pfu caused a lethal infection.

Disease was also examined following inoculation with 7.3 log_10_ pfu virus in older (18-week) adult AG129 mice (N = 8). Virus challenge resulted in rapid disease progression and death (Fig 1A) with an MDD of 5.4 ± 1.6. Although the time to death was significantly later in the 18-week-old mice (p<0.05), the course of disease was similar to young adult mice and included rapid weight loss (Fig 1C) that preceded the onset of clinical signs. Again, none of the animals showed obvious signs of neurologic disease.

### Evaluation of virus dissemination in 703-4-infected AG129 mice

**Fig 2:**
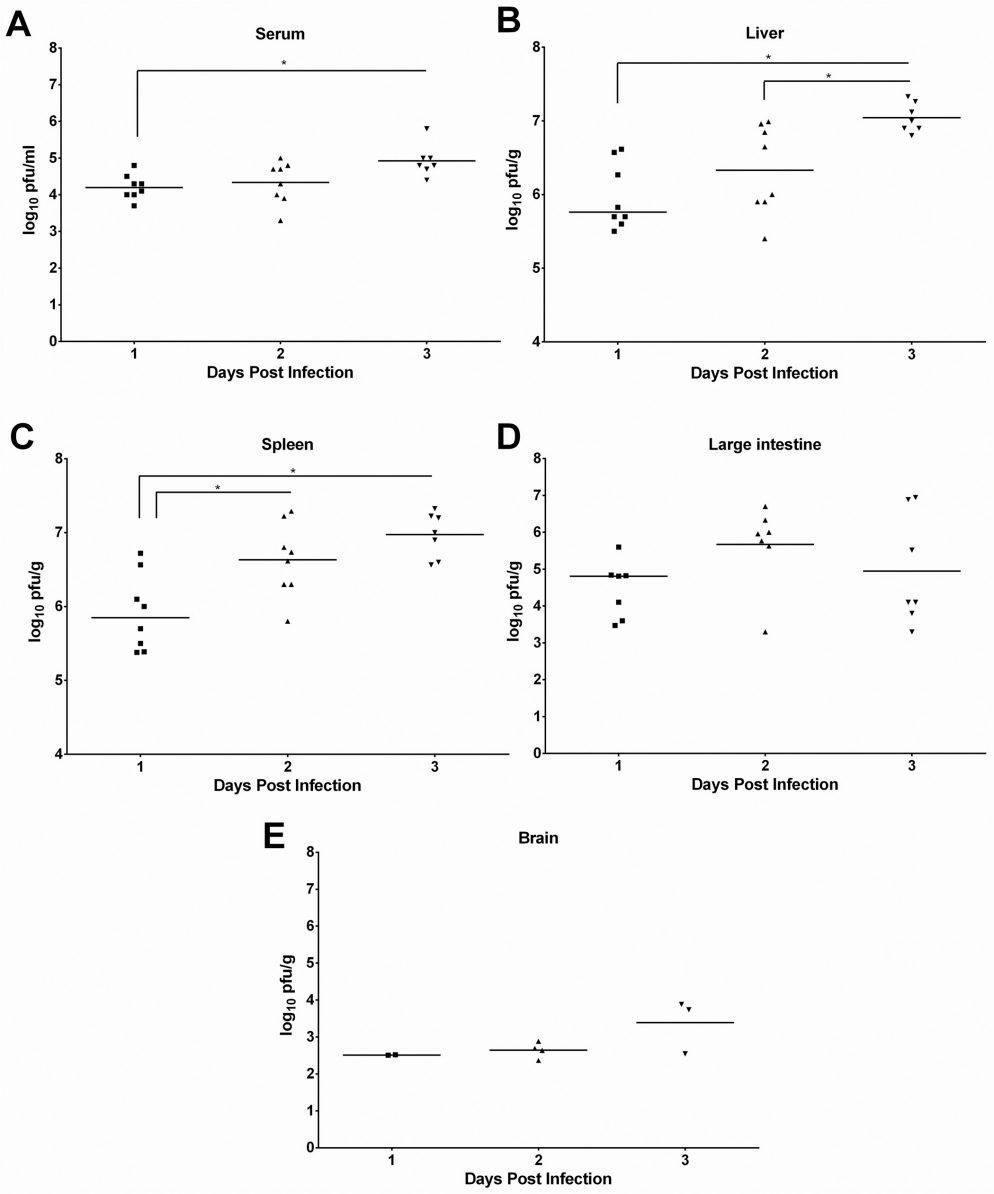
DENV-4 703–4 produces sustained viremia and disseminated infection in multiple organs. Six-week-old AG129 were inoculated with 7.3 log_10_ pfu DENV-4 703–4. On days 1 (n = 8), 2 (n = 8), and 3 (n = 7) post-infection, animals were sacrificed and serum and organs harvested. The organs were weighed, homogenized and virus load in all samples determined by culture. The results shown for (A) serum, (B) liver, (C) spleen, (D) large intestine and (E) brain are combined from two independent experiments. Each symbol represents an individual sample titer. Serum titers are expressed as log_10_ pfu/ml and organ titers as log_10_ pfu/g of tissue. Horizontal lines represent the mean daily titer.; *P<0.05 by ANOVA with Tukey’s multiple comparison test.

To determine the virologic course of DENV-4 703–4 infection, AG129 mice were inoculated with 7.3 log_10_ pfu of the virus as described above. Groups of animals (N = 3-4/day) were sacrificed on days 1, 2, and 3 pi, and serum and multiple tissues (liver, spleen, large intestine and brain) were harvested and viral titers determined by plaque infectivity assays (Fig 2). Additional virus-challenged animals were included in some studies and monitored for weight loss and morbidity as controls to ensure that the course of disease was comparable to that seen in previous studies (data not shown). The study was carried out twice and the data combined for presentation.

Viremia was detected in all animals tested on days 1 (8/8), 2 (8/8) and 3 (7/7) pi. The mean titer on day 1 was 4.2 ± 0.3 log_10_ pfu/ml, which subsequently increased on both days 2 (4.3 ± 0.6) and 3 pi (4.9 ± 0.4) with the increase between days 1 and 3 pi being significant (p<0.05; Fig 2A). Virus was also recovered from both the liver and spleen of all of the animals on all 3 days sampled. Both tissues contained high viral titers, which again rose significantly during the course of the infection between days 1 and 3 pi. The mean liver titer was 6.0 ± 0.4 log_10_pfu/g on day 1, increasing to 6.3 ± 0.6 and 7.0 ± 0.2 on subsequent days (Fig 2B). Spleen titers were 5.9 ± 0.5 log_10_ pfu/g on day 1, increasing to 6.6 ± 0.5 and 7.0 ± 0.3 on days 2 and 3, respectively (Fig 2C). Virus was also recovered from the large intestines of the majority of animals (88% days 1 and 2 and 100% day 3), but the titers were lower than those in the liver and spleen (4.5 ± 0.8 log_10_ pfu/g on day 1, 5.7 ± 1.1 on day 2 and 5.0 ± 1.5 log_10_ pfu/g on day 3; Fig 2D) and there was no significant increase in titer between days 1 and 3 pi. Although virus was detected in the brains of some infected mice, the incidence was lower than in other tissues (38% day 1, 63% day 2, and 57% day 3) and the titers in animals from which virus was recovered were also lower and did not differ significantly as the infection progressed (2.5 ± 0.0 log_10_ pfu/g on day 1, 2.7 ± 0.2 log_10_ pfu/g on day 2 and 3.5 ± 0.6 log_10_ pfu/g on day 3; Fig 2E).

### Effect of DENV-4 703–4 infection on blood chemistry values and cell counts

**Fig 3:**
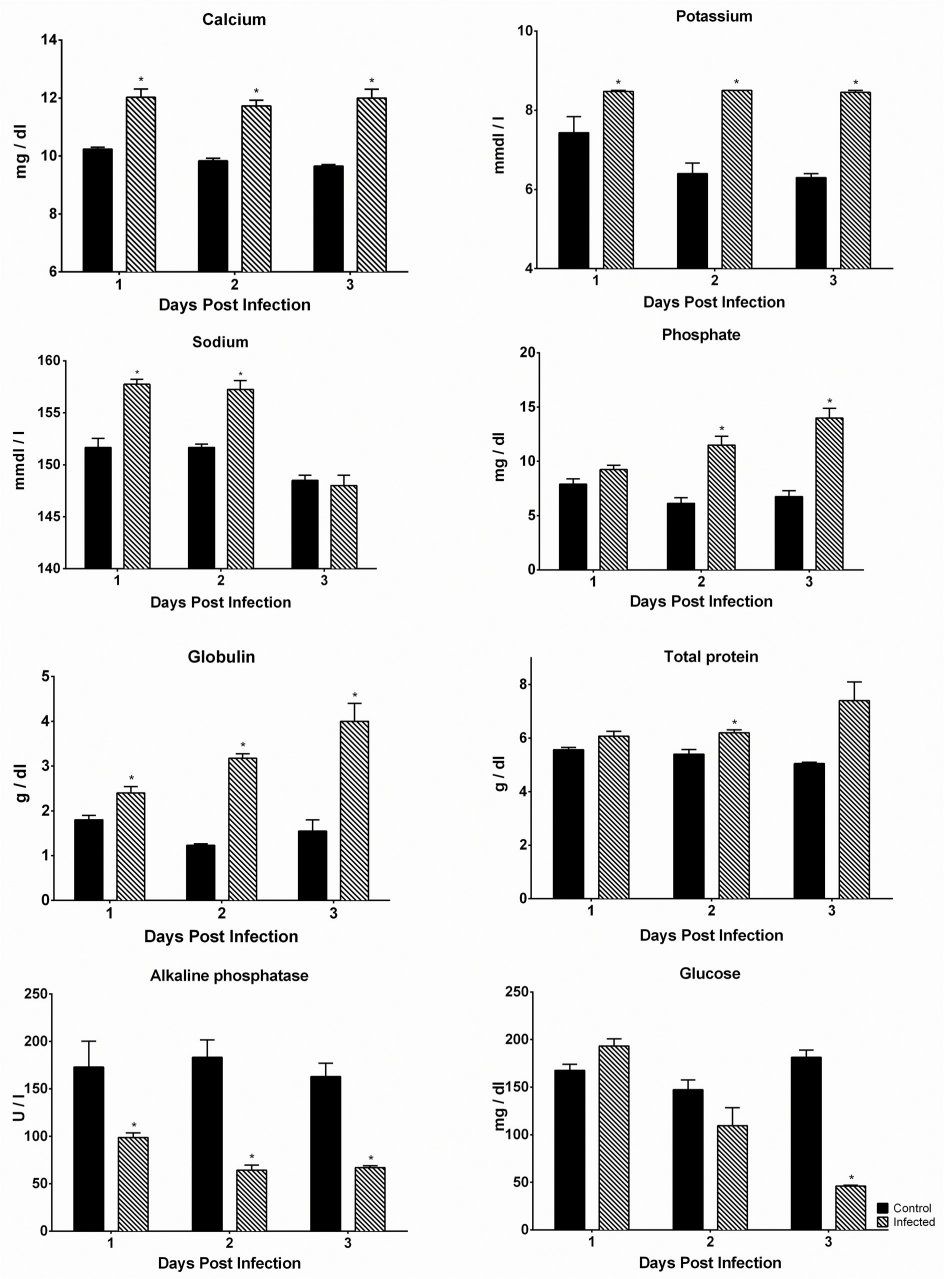
DENV-4 703–4 infection alters blood chemistry profiles. Six week old AG129 were inoculated with 7.3 log_10_ pfu DENV-4 703–4 or an equivalent volume of medium, and blood was collected on days 1, 2 and 3 post-infection. Samples were analyzed using the Vetscan comprehensive diagnostic profile. Bars represent the mean daily value from one experiment performed with groups of 2–4 mice. Asterisks above the DENV-4 703–4 bars are significant (Student’s t test) compared to control values.

To further characterize the infection produced by DENV-4 703–4 in AG129 mice, temporal changes in blood biochemistry profiles were examined. Mice were either mock-infected with medium or challenged with the virus, as described previously, and blood was collected from groups of animals on days 1, 2 and 3 pi. Significant biochemical changes were detected in virus-infected animals beginning as early as day 1 pi, when the animals looked healthy and had only experienced minor weight loss. Levels of calcium (Fig 3A) and potassium (Fig 3B) were both significantly elevated in infected animals on all three days of the study with concentrations of both electrolytes remaining relatively constant temporally. Sodium levels were also significantly elevated in virus-infected animals on days 1 and 2 pi but declined on day 3 pi to levels that were comparable with controls (Fig 3C). In contrast, the impact of infection on serum phosphate levels was detected later with levels only being significantly elevated on days 2 and 3 pi (Fig 3D). In addition to the changes in electrolyte concentrations, serum globulin levels were significantly elevated in virus-infected animals on all of the days tested, and the levels continued to increase as the infection progressed (Fig 3E). Similar results were observed with total protein levels in the blood of virus-infected animals, which increased throughout the study, but were only statistically significant on day 2 pi (Fig 3F). In contrast to the biochemical parameters reported above that were elevated in virus-infected animals, levels of alkaline phosphatase were significantly lower in virus-infected animals than controls on all three days of the study (Fig 3G). Interestingly, blood glucose levels in virus-infected and control animals were comparable on the first two days after virus challenge and it was not until day 3 pi, when infected animals were beginning to show clinical signs of morbidity, that blood glucose levels in virus-infected animals were significantly reduced compared to those in the controls (Fig 3H).

**Fig 4:**
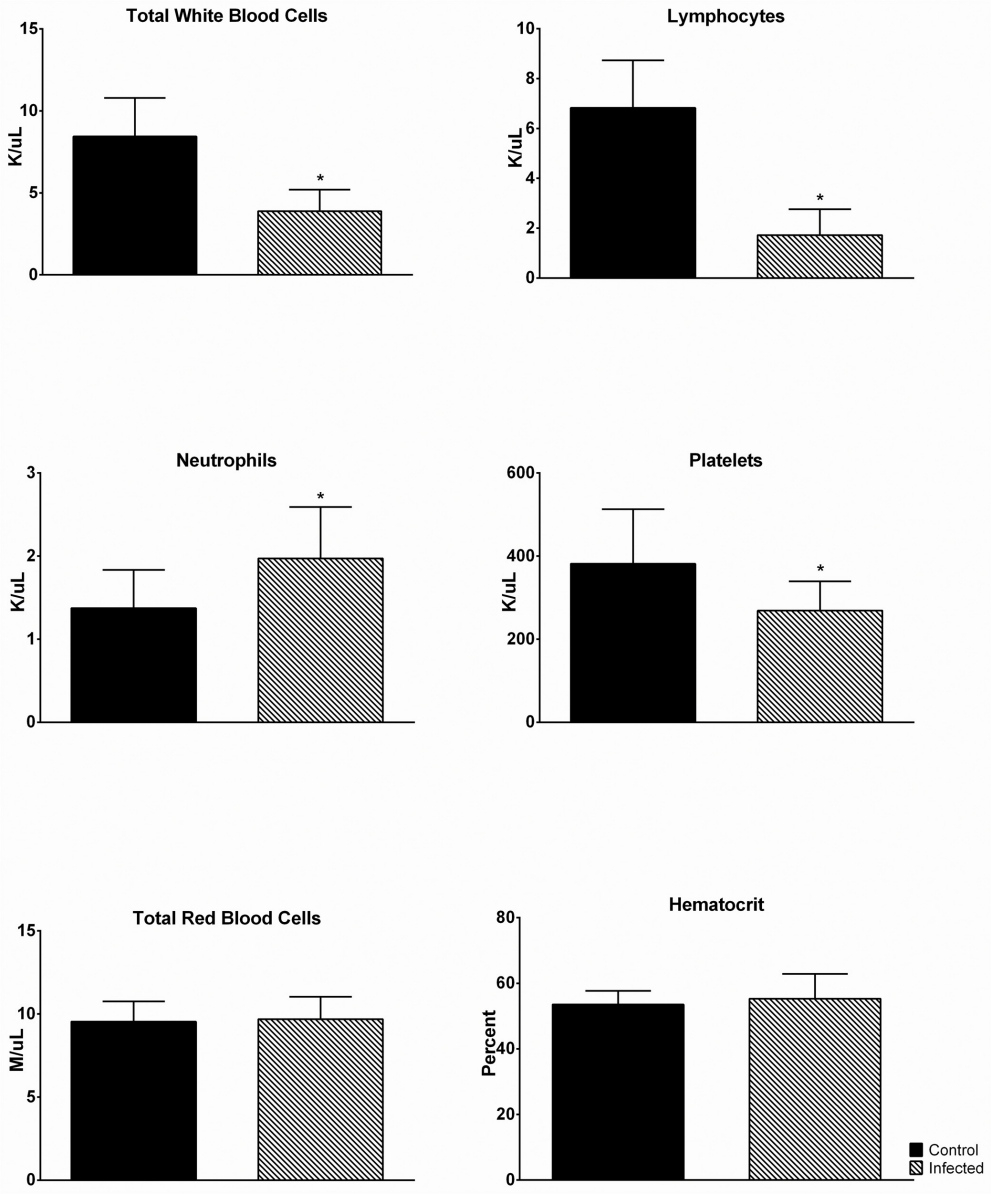
DENV-4 703–4 infection produces leukopenia and thrombocytopenia. Six-to-eight week old AG129 (N = 8) were inoculated with 7.3 log_10_ pfu DENV-4 703–4 or served as uninfected controls (N = 10). Blood was collected on days 1, 2 and 3 post-infection and samples were analyzed using a HEMAVET950TS. Bars represent the mean daily value from one experiment performed with groups of 2–4 mice. Asterisks above the 703-4-infected bars are significant (Student’s t test) compared to control values.

In addition to the biochemical studies we examined the effect of DENV-4 703–4 infection on CBC values. As in previous studies, blood was collected from infected animals on days 1, 2 and 3 pi (N = 3/day) with each animal only being bled once, and cell counts were compared to those from a group of age-matched uninfected control mice (N = 10). Although there were trends towards differences in CBC values for individual days, they failed to reach statistical significance. However, when data for all infected animals was combined across the three days, multiple significant differences between virus-infected and control animals were detected (Fig 4). These included a significant reduction in total white blood cell counts (Fig 4A; p< 0.001) with a corresponding significant reduction in lymphocyte numbers (Fig 4B; p<0.001) but with a small, albeit significant, rise in neutrophil numbers (Fig 4C; p<0.05). Virus-infected mice also exhibited significant thrombocytopenia (Fig 4D; p<0.05). However, both red blood cell numbers (Fig 4E) and hematocrit values (Fig 4F) were comparable in virus-infected and control animals.

### Vascular leakage in the liver and large intestine of DENV-4 703–4 infected mice

**Fig 5:**
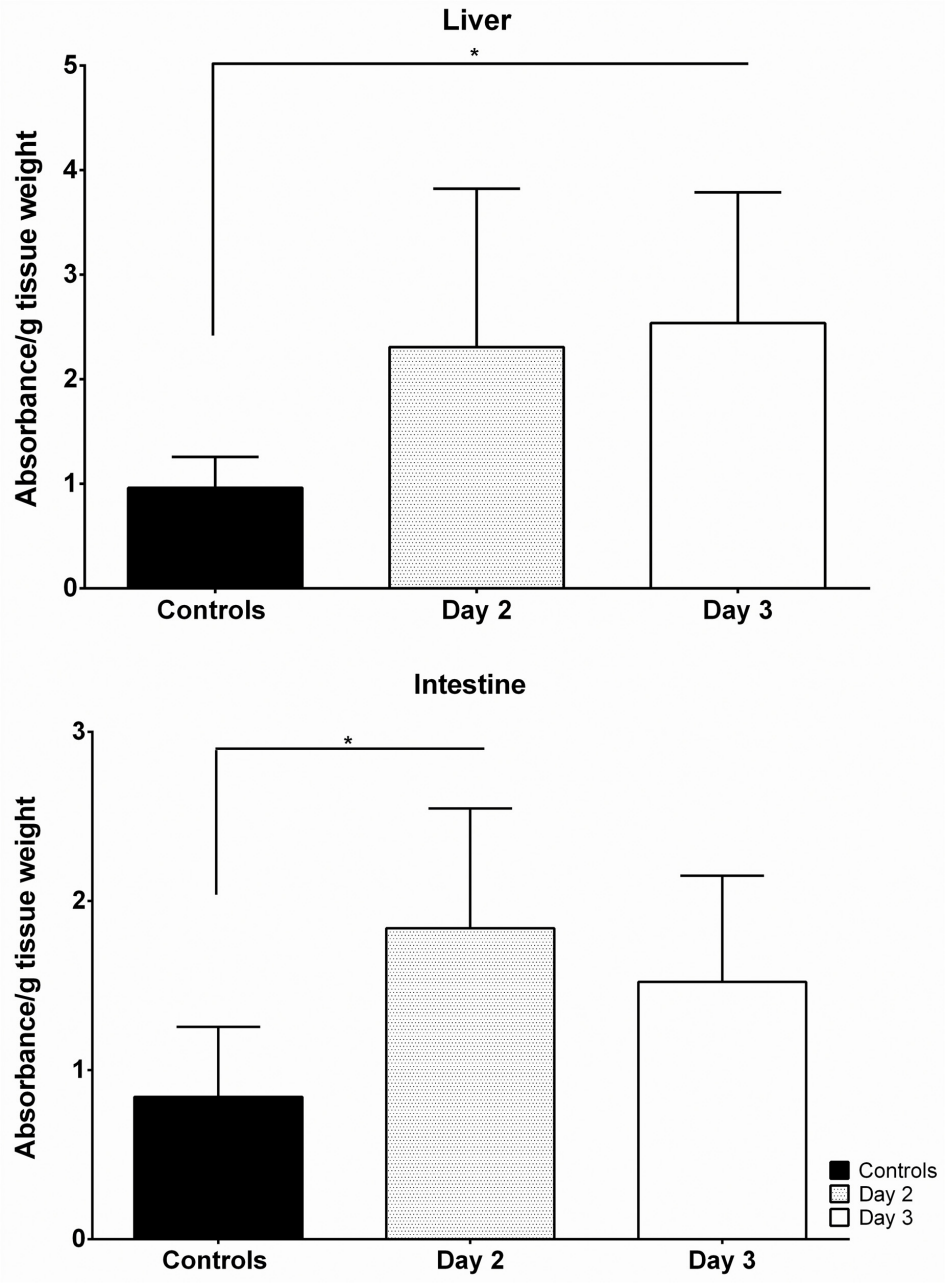
DENV-4 703–4 infection producesvascular leakage. Six-to-eight week old AG129 were inoculated with 7.3 log_10_ pfu DENV-4 703–4 (N = 18) or served as uninfected controls (N = 10). On days 2 (n = 8) and 3 pi (n = 10) animals received 0.2ml of 0.5% Evans blue dye solution by the intravenous route. Two hours later the animals were euthanized and extensively perfused with PBS. Liver and large intestine samples were collected and placed in preweighed tubes with formamide. After 24 hours incubation at 37^°^C the Evans blue dye content of the formamide was measured by absorbance at 610nm.* P<0.05 by ANOVA with Tukey’s multiple comparison test.

Vascular leakage is regarded as a hallmark of dengue infection, including the DENV-2-infected AG129 mouse models [14]. We measured Evans blue dye leakage from the vasculature into the liver and large intestine of DENV-4 infected mice on days 2 (n = 8) and 3 pi (n = 10) to quantitate leakage compared to that seen in uninfected control animals (n = 10). We found that vascular leakage in the liver increased as the infection progressed and was significantly greater than that measured in control animals by day 3 pi (p<0.05; Fig 5).Vascular leakage also increased in the large intestine but the increase was more rapid reaching significance by day 2 pi (p<0.05 each; Fig 5). These results confirmed that vascular leakage was a hallmark of DENV-4 703–4 infection in the AG129 mouse model.

### Altered histopathology in DENV-4 703-4-infected tissues

**Fig 6:**
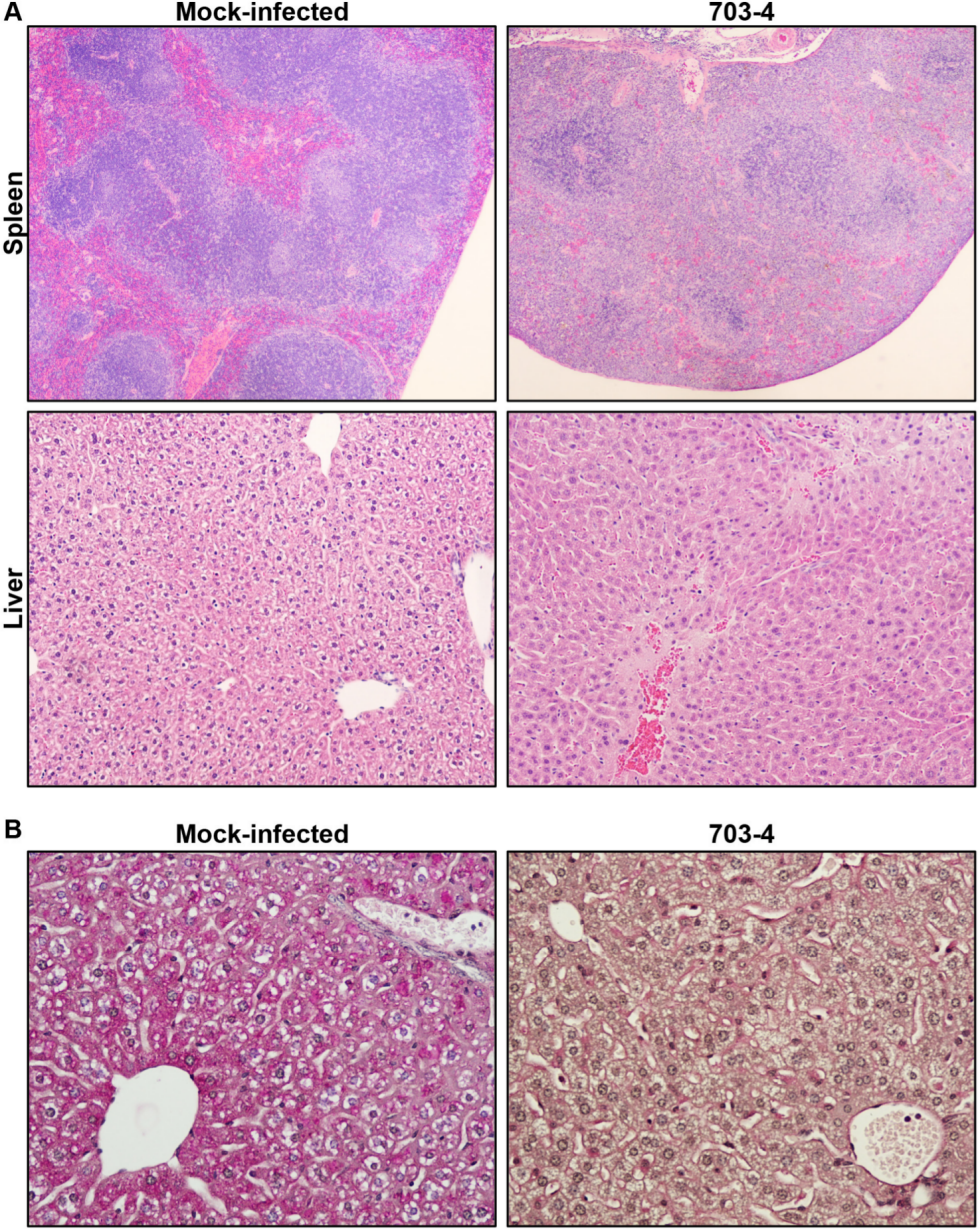
Histological changes produced by DENV-4 703–4 infection. Representative histology images from six-to-eight week old AG129 mice infected with 7.3 log_10_ pfu DENV-4 703–4 or mock-infected controls. (A) Spleen H&E section micrographs (4X magnification) of a mock-infected animal (upper left panel) and a virus-infected animal (upper right panel) showing that the normal white and red-pulp structure is severely altered as a result of infection. Liver H&E section micrograph (10X magnification) from a virus-infected animal (lower right panel) shows areas of focal necrosis and hepatocyte nuclear pleomorphism which are not present in the mock-infected sample (lower left panel). (B) Representative liver section PAS stain micrographs (20X magnification) for hepatocyte content showing depletion in virus-infected tissue (right panel) compared to the red-staining of glycogen in the mock-infected (left panel).

Histological examination of H&E-stained tissue sections was used to study the effect of infection on several tissues. The large intestine and brain of mice infected with 7.3 log_10_ pfu DENV-4 703–4 appeared normal and were indistinguishable from those of naïve and mock-infected animals (data not shown). In contrast, liver and spleen sections from virus-infected animals showed histological changes when compared to mock-infected animals (Fig 6A). The spleens of mock-infected animals contained normally delineated red and white pulp sections; however, the spleens of virus-infected animals showed an overall structural breakdown with increased leukocyte activity and content, and diminished red pulp. Changes were also observed in the virus-infected liver sections. Specifically, the hepatocytes contained pleomorphic nuclei, and several foci of necrosis were observed. In addition, liver sections were examined using Periodic Acid Schiff technique to visualize glycogen in samples from DENV-4 703-4-infected animals, and results showed depletion of glycogen stores in hepatocytes (Fig 6B). Immunohistochemistry was not possible in these studies as commercial anti-DENV-2 NS3 antibody did not recognize DENV-4.

### Impact of DENV-4 703–4 infection on cytokine and chemokine responses

**Fig 7:**
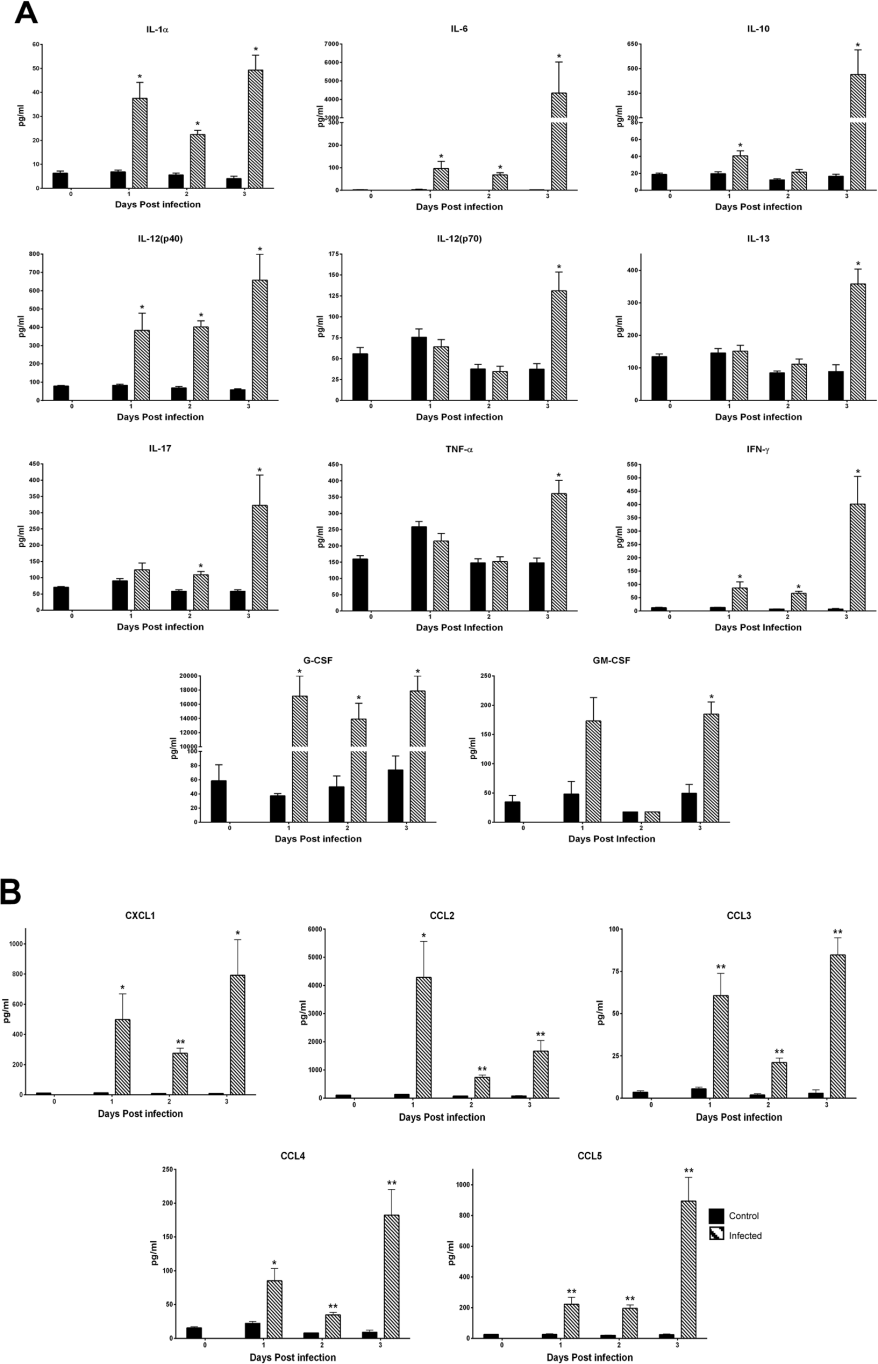
DENV-4 703–4 infection results in increased serum levels of cytokines and chemokines. Serum was collected from DENV-4 703-4-infected (n = 7) or media-treated control mice (n = 5) on the indicated days post infection and levels of cytokines (A) and chemokines (B) were quantified from a panel of 23 cytokines using a multiplex bead assay (Bio-Rad, Hercules, CA) following the manufacturer’s protocols. Results shown are pooled from two separate experiments Cytokine abbreviations are as follows: IL: interleukin, KC: keratinocyte chemoattractant, G-CSF: granulocyte-colony stimulating factor, IFNγ: interferon gamma, TNFα: tumor necrosis factor alpha, MCP-1 (CCL2): monocyte chemoattractant protein-1 (CCL3 or 4), MIP: macrophage inflammatory protein, RANTES (CCL5): regulated on activation, normal T cell expressed and secreted, GM-CSF: granulocyte/macrophage-colony stimulating factor. * P<0.01, ** P<0.001 (Student’s t test).

The innate immune response to DENV-4 703–4 infection was examined in AG129 mice inoculated with 7.3 log_10_ pfu of the virus or an equal volume of tissue culture medium. Animals (N = 3-4/group/day) were sacrificed on days 1, 2, and 3 pi and serum collected and analyzed by Bioplex assay. Virus infection resulted in significantly elevated levels of a number of cytokines and chemokines (Fig 7). In general, the production of cytokines and chemokines increased from baseline levels during the infection and peaked on day 3 pi although levels of the chemokine CCL2 (MCP-1) peaked on day 1 pi and decreased thereafter. Levels of cytokines such as IL-1α, IL-6, IL-10, IL-12p40, IFN-γ, and G-CSF were significantly higher in virus-infected animals compared to mock infected controls beginning on day 1 pi. Levels of other cytokines such as IL-12 p70, IL-17, IL-13 and TNF-α were not significantly higher than mock-infected controls until day 3 pi. Levels of CXCL1 (KC), CCL2 (MCP-1), CCL3 (MIP-1α), CCL4 (MIP-1β) and CCL5 (RANTES) chemokines were significantly higher than mock-treated controls on days 1–3 pi.

### Antibody-dependent enhancement (ADE) of sublethal DENV-4 703–4 infection

**Fig 8:**
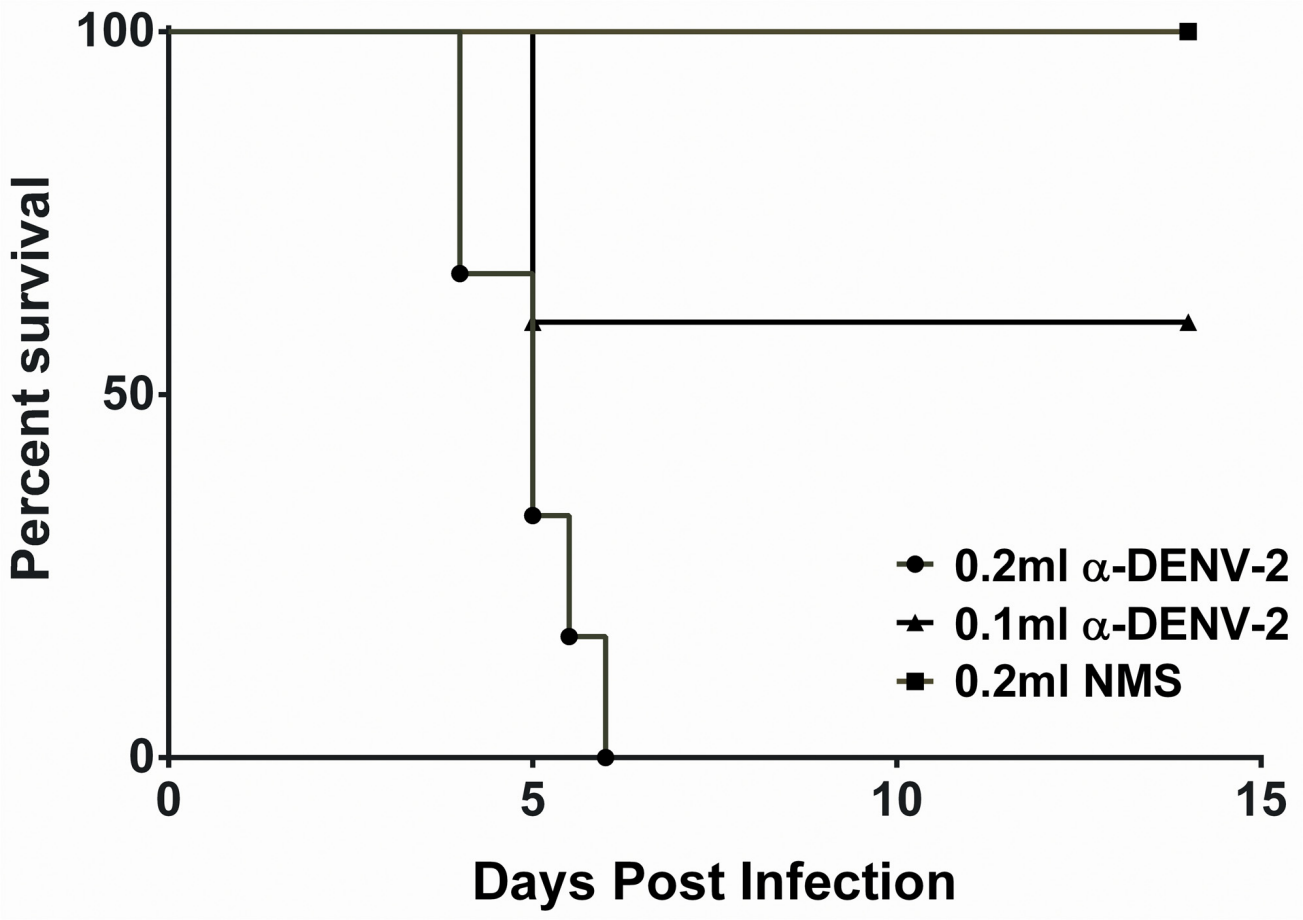
Lethal enhancement of DEN-4 703–4 disease by anti-DENV sera. Kaplan-Meier survival curve for 6–8 week old AG129 mice (N = 6-7/group) administered 0.1 ml (▲) or 0.2ml (●) of anti-DENV serum or 0.2ml normal mouse serum (■) one day prior to iv challenge with 6.3 log_10_ pfu DENV-4 703–4.

Lastly, we evaluated the ability of a sub-lethal inoculum of DENV-4 703–4 to produce a lethal infection under conditions of ADE. Groups of mice (N = 6–7) were administered mouse anti-DENV-2 PL046 serum or normal mouse serum (NMS) one day prior to a sub-lethal dose of 6.3 log_10_ pfu of DENV-4 703–4. Mice were observed twice daily for morbidity and mortality, and the survival curve is shown in Fig 8. All of the animals (6/6) that received 0.2 ml of anti-DENV-2 (PL046) serum experienced disease with progression to death by day 6 pi. All (6/6) control animals that received 0.2 ml of normal mouse serum survived and remained healthy throughout the study. In the group of mice pre-treated with 0.1 ml of anti-DENV-2 serum there was an intermediate phenotype with 2/6 animals dying by day 6 pi and all animals experiencing morbidity. Therefore the anti-DENV-2 polyclonal serum enhanced DENV-4 703–4 infection with a dose-dependent effect of antibodies causing the enhancement.

## Discussion

The initial description of a lethal model of DENV-2 in AG129 mice [13] was an important achievement for dengue research and for the evaluation in a small animal model of candidate vaccines and therapeutics against this globally important mosquito-borne disease. Subsequently, the relevance of the model was greatly increased by the development of mouse-adapted DENV-2s that produce a lethal disseminated disease rather than a chiefly neurologic infection [14, 15, 25] and by the identification of a non-mouse-adapted DENV-2 that can produce disseminated lethal disease [21]. However, the lack of comparable models of the other three DENVs has limited the potential applications of the model. This has begun to change recently. Two models of lethal DENV-4 infection in AG129 mice have recently been reported [26]. Both of these models utilized viruses that had been mouse-adapted, but using two different approaches. In the first, newborn AG129 mice were inoculated with DENV-4 strain H-241 and a clarified brain homogenate was prepared from those pups that became moribund. Young adult animals were inoculated with this material and another brain homogenate prepared from moribund animals. After two additional *in vivo* passages the final clarified brain homogenate was passaged *in vitro* in C6/36 cells to prepare the virus stock used in challenge studies. The second method was similar to that used to derive DENV-2 strain D2S10 [14]. DENV-4 TVP-376 was passaged by alternating rounds of *in vitro* amplification and *in vivo* infection. Specifically, C6/36 cells were infected, and the virus obtained was concentrated by ultracentrifugation over a glycerol cushion. This preparation was subsequently inoculated into young adult AG129 mice, and serum was collected four days later to use as inoculum for another round of *in vitro* amplification [26]. Both methods of mouse adaptation resulted in models with high lethality, but detailed characterization studies were not reported.

We developed the DENV-4 strain 703–4 model in AG129 mice described here by identifying a low passage non-mouse-adapted virus strain that could replicate to high titer in C6/36 mosquito cells and then challenged young adult AG129 mice with high titer inocula. These studies showed that an inoculum of ≥10^7^ pfu of virus produced a rapidly lethal infection with the animals dying between days 3 and 5 post challenge. However, the model has a limited dynamic range, because a 10-fold reduction in inoculum (from 7.3 log_10_ pfu to 6.3 log_10_ pfu) resulted in loss of lethality The inoculum required to produce lethal infection was similar to that used in the DENV-2 model for the mouse-adapted D2S10 virus [14] but was significantly more than the 5 x 10^4^ or 1 x 10^5^ pfu of DENV-4 required to produce a lethal infection by Sukupolvi-Petty et al [26], and 10^4^ pfu for DENV-2 D2Y98P [21, 22].

Our initial studies were conducted in young adult mice (6–8 week old), which are suitable for many studies, including evaluation of the efficacy of putative antiviral drugs and other therapeutics. However, efficacy evaluation studies for vaccines typically require the ability to produce outcome in older animals due to the need for administration of one or more doses of a candidate vaccine and for time for the immune response to develop prior to challenge. Accordingly, we evaluated the ability of DENV-4 703–4 to produce disease in older animals (18 weeks). We observed no reduction in disease severity in the older animals indicating that this model is suitable for testing vaccine candidates.

In the current studies, inoculation of AG129 mice with DENV-4 703–4, like previous studies with DENV-2 D2S10, produced a rapidly progressive infection and direct titration of isolated tissues demonstrated virus dissemination to multiple organs. Viremia was detected in all animals tested beginning on day 1 pi with titers increasing on subsequent days. Sustained virus replication was also observed in the liver and spleen of all animals and in the large intestine of >90% (21/23) of animals tested, although the variability in titer between animals in the intestine was larger than in the other tissues. The high viral titers in the spleen and large intestine that we observed are similar to the results seen previously in studies in AG129 mice inoculated with a firefly luciferase-expressing DENV-2 16681 in which lymphoid tissues and the intestines were identified as major sites of virus localization by bioluminescent imaging [27]. We also detected virus in the brains of some infected mice. The overall virus detection rate in the brain was 39% (9/23) across the three days with the highest incidence occurring on day 2 pi in 56% (5/9) of the animals. However, viral titers in brain were much lower than in sera and in other tissues sampled. Interestingly, although time-course studies were not reported with strain D2S10, virus was recovered from the brains of 100% (7/7) of mice tested on day 3 pi [14], indicating that brain viral loads from 703-4-infected mice were similar to the DENV-2 models of disseminated disease. Further, no clinical signs or histopathological changes consistent with neurologic disease were observed after DENV-4 703–4 infection. This suggested that, virus that reached the brain was not responsible for the rapid progressive disease. Dengue is typically considered a disseminated rather than neurologic disease; however, neurologic signs and encephalitis are reported in a number of dengue patients [28].

Although no histologic changes were detected in the brains and intestines of virus-infected mice, the splenic architecture was disrupted, and the livers contained necrotic foci. Hepatocytes had pleomorphic nuclei and diminished glycogen stores; these observations are consistent with the observed deterioration in condition of the animals. Overall, the histology results were similar to those reported with DENV-2 strains in AG129 mice. Generally, infection with DENV-2 was more severe due to the presence of inflammatory infiltrates and intestinal damage. [14, 21, 29].

Vascular leakage, a hallmark of dengue infection, was demonstrated in the liver and large intestine of DENV-4-infected animals. AG129 mice infected with DENV-4 703–4 developed multiple CBC changes, most notably a significant thrombocytopenia, which is known to be an important clinical finding associated with dengue [2, 30]. The infected animals also had significant leukopenia, which appeared to be attributable to a drastic decrease in lymphocytes, whereas neutrophils were significantly elevated. The infected animals also experienced multiple alterations in blood chemistry values. A number of the changes can be associated with damage to individual organs, in particular the kidney (elevated calcium and potassium). However, they are also associated with dehydration. Taken together, the biochemical changes coupled with observed weight loss, and clinical signs are strongly suggestive of progressive dehydration and metabolic collapse. Reduced food and water intake as the animals develop clinical signs likely contribute to this process. We had anticipated that the animals would experience hypovolemia due to plasma leakage, which would again closely mimic the clinical situation [2, 30]. However, CBC results did not show elevated red blood cell counts or increased hematocrit values that were observed in AG129 mice infected with DENV-2 strain D2Y98P [21, 22].

High levels of multiple pro-inflammatory cytokines have been detected during severe dengue in humans. These consistently include IFN-γ, TNF-α, IL-6 and IL-10, which are believed to be important because they may contribute to disease pathogenesis, particularly to increased vascular permeability [8, 30]. The original report of the severe dengue produced by DENV-2 D2S10 in AG129 mice demonstrated the importance of TNF-α in disease progression [14]. Subsequently, elevated levels of TNF-α, IFN-γ and, IL-6 were reported in DENV-infected AG129 mice following inoculation with the non-mouse-adapted DENV-2 strain D2Y98P [21], and all four cytokines were also shown to be elevated in ADE models of severe DENV-2 disease [15, 17, 25]. Furthermore, in the current studies AG129 mice inoculated with DENV-4 703–4 experienced significantly elevated levels of multiple cytokines and chemokines, including the four discussed above, providing further evidence for their central role in dengue pathogenesis. Interestingly, DENV-4 703–4 infected mice displayed an increase in TNFα on day 3 pi compared to mock-infected controls, and DENV-2 D2S10 resulted in increased TNFα levels on day 3 pi compared to the parental DENV-2 strain PL046, not mock-infected controls. Also, the studies with DENV-2 D2Y98P did not include mock-infected controls but showed a time-dependent increase in TNFα Thus, direct comparison of these different models is challenging. However, it is clear that the increased level of TNFα is a consequence of infection by both DENV-2 and DENV-4 viruses.

ADE is used for mouse models of severe dengue for a variety of reasons. Many investigators believe that ADE models more closely mimic the biology associated with clinically severe DEN [7]. In addition, most AG129 mouse models of dengue require high-titer challenge to produce lethality; thus, one practical advantage is that ADE reduces the quantity of virus required to induce disease in the AG129 mouse model. In the current studies, DENV-4 703–4 infection of AG129 mice could be enhanced using mouse anti-DENV-2 serum. Interestingly, enhancement of DENV-4 703–4 required 0.2 ml of anti-DENV-2 serum to produce 100% lethality, whereas enhancement of DENV-2 to produce a 100% lethal infection required as little as 0.025 ml of anti-DENV-1 serum [17], suggesting either that the non-mouse-adapted DENV-4 model is not as virulent as DENV-2 D2S10 [17], or that the requirement for cross-reactive antibodies for enhancement is distinct for DENV-4.

In summary, this report describes a new AG129 mouse model of lethal infection produced by a non-mouse-adapted Thai DENV-4 strain. The model is comparable to those previously reported for DENV-2. Specifically, high-titer virus challenge was required to produce lethality. Following challenge, the virus disseminates and replicates in multiple visceral tissues. The infection shows many virologic, immunologic and biochemical similarities to human dengue and suggests that the mechanism of disease by DENV-4 is similar to that in DENV-2 models. However, some characteristics of the DENV-4 model indicate that it may be less virulent than viruses used in the DENV-2 models, including a weaker capacity to cause ADE and no intestinal tissue damage or inflammation. One possible explanation for these differences is that DENV-4 strain 703–4 is a natural DENV-4 isolate that has not been subjected to either multiple cell culture or mouse passages. Ultimately, this model is an important addition to the field of dengue research, particularly in understanding similarities and differences in the pathologic basis of the disease caused by different viral serotypes and in determining comparative efficacy of therapeutic interventions.
